# The economic and societal burden associated with adolescent idiopathic scoliosis: A burden-of-disease study protocol

**DOI:** 10.1016/j.xnsj.2023.100231

**Published:** 2023-05-18

**Authors:** Thomáy-Claire Ayala Hoelen, Paul C. Willems, Jacobus J. Arts, Ghislaine van Mastrigt, Silvia Evers

**Affiliations:** a Department of Orthopedic Surgery and CAPHRI Research School, Maastricht University Medical Center (MUMC+), P.Debyelaan 25, Maastricht, 6202AZ, The Netherlands; b Department of Health Services Research, Faculty of Health, Medicine and Life Sciences, CAPHRI, Maastricht University, , Universiteitssingel 40, Maastricht, 6229 ER, The Netherlands; c Trimbos Institute, Netherlands Institute of Mental Health and Addiction Utrecht, 3521 VS Utrecht, The Netherlands

**Keywords:** Scoliosis, Costs and cost analysis, Burden of disease, Cost of illness, Societal perspective

## Abstract

**Background:**

Adolescent idiopathic scoliosis (AIS) has an estimated general population prevalence of 2% to 3%. The impact of adolescent idiopathic scoliosis (AIS) on the patients’ experienced quality of life and psychological well-being and the resulting societal burden are increasingly recognized. However, there is limited knowledge on the economic burden of AIS. This cross-sectional, prevalence-based, bottom-up approach burden of disease study aims to determine the impact associated with adolescent idiopathic scoliosis in terms of the cost-of-illness and health-related quality of life from a societal perspective in the Netherlands.

**Methods:**

Persons diagnosed with AIS or parents of a child with AIS that are willing and able to answer the questionnaires will be eligible to participate. Patients will be included consecutively between June until January 2023. Costs and self-perceived health-related quality of life will be estimated using 3 steps: identification, measurement and valuation. To assess the costs associated with AIS the institute for Medical Technology Assessment - Medical Consumption Questionnaire and the institute for Medical Technology Assessment – Productivity Cost Questionnaire will be used. To assess the HRQoL of adult AIS patients the EuroQol 5-dimensions or EuroQol 5-dimensions Youth questionnaire for children under the age of 12 and the Scoliosis Research Society-22 revised questionnaire will be considered.

**Discussion:**

This is the first study in this field. It will help raise awareness for AIS and wider support for both the patient community and informal care takers among healthcare professionals and policymakers. Major strengths of this study will be the use of mostly validated, standardized questionnaires. Limitations include the cross-sectional and retrospective nature of the study design.

## Background

Adolescent idiopathic scoliosis (AIS) refers to a 3-dimensional deformation of the spine in children aged 10 to 18 years old [1]. The term idiopathic refers to the etiology of AIS, which is unknown but suspected to be multifactorial [2]. AIS predominantly affects females and the general population prevalence varies in literature but is most commonly reported to be around 2% to 3% [1]. According to the Scoliosis Research Society, AIS can be diagnosed if the Cobb angle of the spinal curve on the frontal standing radiograph is equal to or exceeds 10° and axial rotation is present [1]. AIS can be managed conservatively for mild to moderate curves using physiotherapy and/or bracing, as well as surgically in case of severe curve progression [3], [4].

The scoliotic curves tend to progress most rapidly during the growth-spurt in adolescence before stabilizing when skeletal maturity is reached [5]. Severe curve progression can lead to serious health detriments such as cardiovascular complications, impaired pulmonary function, chronic pain and psychological strain [6], [7]. The impact of AIS on the experienced quality of life and the psychological impact of AIS are increasingly recognized [1], [8], [9]. Gallant et al. [10] reported that patients with AIS have significant issues with body image perceptions and have a higher risk of developing mood disorders.

Correction of the scoliotic curve can significantly improve body image perception [4], [11]. However, surgical correction is not without risk. Surgical complications are reported in 5% to 25% of the cases with the most severe complication being neurological injury [4], [12]. Additionally, scoliotic curve correction is considered a costly surgical intervention, with facility costs comprising the largest share [13], [14].

Patients with scoliosis consume a substantial amount of healthcare resources based on their health-related quality of life regardless of the severity of clinical symptoms [15], [16]. Glassman et al. [16] reported that the estimated mean nonoperative treatment costs over a 2-year observation period were higher for patients with more severe symptoms. Additionally, Glassman reported in an earlier study that patients with more severe clinical symptoms were more likely to be disabled and unemployed [15]. This poses a supplementary societal as well as an economic burden on an already burdened healthcare system. Thus, early detection and treatment are crucial in minimizing the impact of AIS and thus reducing potential surgical costs as well as mitigating long-term healthcare outcomes. One potential strategy for early detection is institutionalized screening for example by a school doctor. Yet, there is no consensus in literature on the benefits of screening that is, early detection and thereby reduction of adverse health outcomes and costs [5].

In general, there are limited publications available on the economic burden and health-related quality of life of patients with AIS. Moreover, to the best of our knowledge, no studies have been published in the Netherlands. More insight will contribute to the ongoing discussion regarding the potential relevance of early detection and adequate treatment to help mitigate the societal burden of AIS. Therefore, this cross-sectional, prevalence-based, bottom-up burden of disease study aims to determine the associated impact of adolescent idiopathic scoliosis in terms of the societal costs and generic health-related quality of life in the Netherlands.

## Methodology

### Study design

This cross-sectional, prevalence-based, bottom-up approach burden of disease study will adopt a societal perspective. Cross-sectional nature of the study implies that measurements will be done at one point in time [17]. Prevalence-based studies consider the costs attributable to the total number of cases in a set time frame (usually a year). On the contrary, incidence-based studies refer to the number of new cases that arise within a set time frame and attempt to estimate the life-time costs. A societal perspective means that all costs regardless of who incurred them will be considered.

This study will aggregate data at the population level from 2 questionnaires considering costs and 2 questionnaires considering the self-perceived health-related quality of life. The study will be conducted at the Maastricht University Medical Centre (MUMC+). Non-WMO approval has been obtained from the Medical Ethical Testing Committee (METC) of the University Maastricht/ academic hospital Maastricht (UM/MUMC+) as well as consent from the advisory board of the MUMC+ (METC 2022-3166). Informed consent will be obtained from all participants before the start of the study. The study will follow the Dutch guidelines for costing studies. It will be reported in accordance with the Consolidated Health Economic Evaluation Reporting Standards (CHEERS 2022) to enhance reporting quality and transparency and takes the guidelines published by Larg and Moss into account [18], [19], [20].

### Participants and procedure

All persons diagnosed with AIS or parents of a child with AIS in case a child is unable to fill out the questionnaire, that is willing and able to answer the questionnaires will be eligible to take part in this study. Participants diagnosed with other types of scoliosis, who do not reside in the Netherlands or those who are unable to understand the Dutch language will be excluded.

The digital questionnaire will be distributed using social media, mail and via QR codes distributed to relevant medical professionals in the Netherlands for example, orthopedic surgeons, physician assistants, physical therapists and Schröth therapists working with AIS patients. Furthermore, the Dutch scoliosis patient society and Stichting I love my back will be requested to distribute the digital questionnaire among its members via e-mail and social media. Paper versions of the questionnaires or telephone consultation will also be available upon request. Despite the lack of a standard sample size calculation methodology, a sample of at least 100 participants will be aimed to get enough variation in the patient population. Patients will be included consecutively between June and January 2023.

### Data collection

All data will be collected between June and January 2023. The questionnaires consist of generic questions regarding patient demographics such as age and gender as well as health and cost-specific questions. The questionnaires will be distributed using the online survey tool Qualtrics [21]. The option “prevent multiple submissions” in Qualtrics was opted for to limit participants from submitting multiple responses. The questionnaires will be tested in a pilot study of (n=5) among participants that will not be included in the final study. Obtained qualitative feedback will be incorporated to finalize the questionnaires.

### Cost-estimation

A bottom-up costing approach will be used whereby cost estimation will be made using 3 steps: (1) identification, (2) measurement and (3) valuation, to estimate the associated costs with AIS [17], [22].

### Identification of costs

According to the patient's pathway, cost categories will be used to help identify and structure relevant costs. Health sector costs will include costs related to the diagnosis and treatment of AIS such as costs for X-rays, consultations, and surgical interventions. Any costs incurred or contributions made by the patient or family such as informal care or transportation costs to and from the hospital will fall under patient and family costs. Productivity losses made by the family for taking days of work or unemployment resulting from AIS will be considered. The final category includes costs such as the loss of schooldays/absence from school. An overview of the cost categories and items that will be considered are presented in Table 1.

**Table 1:** Cost items.

Item			
Healthcare sector costs	Patient & family costs	Productivity losses	Other sector costs
General practitioner	Transportation	Absenteeism	Loss of school days
Social Worker		Presenteeism	
Physical Therapist			
Occupational therapist			
Speech therapist			
Dietician			
Homeopath/acupuncturist			
Psychologist/psychiatrist			
Occupational physician			
Inpatient care (overnight hospital stay)			
Day treatment			
Outpatient care (<24 hrs)			
Emergency care			
Medication			
Home care			

### Measurement of costs

To assess the costs associated with AIS the institute for Medical Technology Assessment - Medical Consumption Questionnaire (iMTA-MCQ) and the institute for Medical Technology Assessment – Productivity Cost Questionnaire (iMTA-PCQ) will be used [23]. The iMTA-MCQ is a generic instrument that measures medical consumption. It consists of 36 questions and considers a prior period of 3 months. The iMTA-PCQ consists of 18 questions and aims to assess all aspects concerning productivity losses for example, absenteeism, presenteeism and productivity losses related to unpaid work [24]. The questions of the iMTA-PCQ concern a prior period of 4 weeks to limit recall bias. Further, an assessment of the validity and reliability of the iMTA-MCQ and the iMTA-PCQ still needs to be conducted.

### Valuation of costs

Data on health care consumption was obtained from the iMTA-MCQ and productivity costs were obtained from iMTA-PCQ [23]. Considering the variation in recall time the questionnaires refer to (ie, iMTA-MCQ: 3 months, iMTA-PCQ: 4 weeks), results will be extrapolated to 12 months. The Dutch guidelines for pricing existing costs will be used [19]. The unit cost per item will be calculated based on the cost per item and multiplied by the volume [19]. The existing costs consist of prices such as the cost of healthcare consultations and/or medication [25]. In case of medication cost data, the lowest cost price will be used. Additionally, a delivery cost will be added to the cost of prescribed medication.

As described by the Dutch Costing Guidelines productivity losses will be calculated based on the total time of absenteeism multiplied by the regular cost of an employee, using the Friction Cost Method (FCM). FCM assumes that after a friction period (±12 weeks) an absent employee will be replaced [19]. Although, a friction period of 12 weeks is advised in the Dutch Costing Guidelines, a friction period will be calculated reflecting the labor market in 2022 in the Netherlands. In line with the Dutch Costing Guidelines informal care will be calculated using the replacement cost method [19]. Inflation of prices is considered, and costs will be indexed to the year 2022 using rates from the Dutch Central Bureau for Statistics [26]. All costs will be presented in euros.

## Self-perceived health-related quality of life assessment

### Identification of generic health-related quality of life (HRQoL)

Health-related quality of life (HRQoL) can be used to determine the burden of disease on physical, mental health, social functioning and well-being of an individual [22]. To assess the HRQoL of AIS patients the EuroQol 5-dimensions (EQ-5D-5L) or EuroQol 5-dimensions Youth (EQ-5D-Y) questionnaire and the Scoliosis Research Society-22 (SRSr-22) revised questionnaire will be considered.

### Measurement of HRQoL

The EQ-5D-5L is a generic questionnaire assessing the overall health status of a patient. Generic questionnaires allow for comparison of outcomes across populations and interventions since they are not disease-specific but rather adopt a more general perspective [27]. The EQ-5D-5L is commonly used and recommended for economic evaluation [17], [19]. It consists of 5 domains each consisting of 1 question: (1) mobility, (2) self-care, (3) pain/discomfort, (4) usual activities and (5) anxiety/depression. Each question is scored using a 5-level scale consisting of the options: no problems, slight problems, moderate problems, severe problems, and extreme problems/inability to complete a task.

Additionally, the questionnaire consists of a visual analogue scale (EQ-VAS) assessing the patient's self-reported health-status. The EQ-5D-5L questionnaire was developed to outperform its predecessor the EQ-5D-3L questionnaire which only has 3 answer options per domain. The EQ-5D-5L appears to perform better in terms of ceiling effects when compared to the EQ-5D-3L questionnaire indicating that fewer people reported the health status “11111” which indicates full health [28]. However, ceiling effects are most common in the domain of self-care which is a relevant domain for patients with AIS and thus should be considered a limitation [29]. Overall, the EQ-5D-5L has been established to have adequate psychometric properties since it is a valid and reliable tool in addition to having acceptable responsiveness [28], [29].

Participants younger than 15 years old were provided with the EQ-5D-Y instead of the EQ-5D-5L questionnaire. The EQ-5D-Y is a child-friendly EQ-5D questionnaire that is based on the EQ-5D-3L version. It consists of the same 5 domains as the EQ-5D and each question is scored using a 3-level scale for example, no problems, some problems and a lot of problems.

In addition to the EQ-5D-5L, a disease-specific quality of life questionnaire will be provided namely the SRS-22r [30], [31]. Disease-specific questionnaires are designed for specific patient populations making them pertinent to measuring aspects that are not covered in generic questionnaires but are relevant in capturing the HRQoL of specific patient populations [27], [32]. However, they are less compatible for comparison to other diseases or populations [27].

The SRS-22r is a self-assessed questionnaire and consists of 5 domains: (1) function, (2) pain, (3) mental health, (4) self-image and (5) management satisfaction/dissatisfaction. The SRS-22r includes a total of 22 questions divided among these 5 domains (each domain has 5 questions apart from the fifth domain consisting of 2 questions). The scoring of each question ranges from 1 (worst) to 5 (best), with a maximum score of 110. The methodological quality of the Dutch translated SRS-22r version was excellent [30], [33]. The Dutch SRS-22r version indicated fair methodological quality (ICC ≥ 0.70) but showed ceiling effects for the domains function, pain and management satisfaction/dissatisfaction. However, no cross-cultural validation was performed implying that it has not been tested whether the SRS-22r that was originally developed in as the SRS-24 in the United States is applicable and equivalent to the Dutch culture [33], [34].

### Valuation of HRQoL

The answer to the EQ-5D domains can be summed to provide a total of 3,125 health condition states [28]. Subsequently, these health conditions can be assigned an index or utility value based which can be used for economic evaluations. Utility scores are specific for the Netherlands and based on prior studies that used a general population sample to determine preference weights for hypothetical health conditions [29], [35]. The EQ-5D-5L utility scores will be used to compute a QALY score [35]. This is achieved by multiplying the utility scores by the duration of time spent in that health state [36]. The SRS-22r instrument will be used to address AIS specific effects on the self- perceived health-related quality of life that are not included in the generic EQ-5D-5L instrument.

### Statistical analyses

The normality assumption is usually violated for the cost data, therefore nonparametric bootstrapping will be performed (1,000 replications) per cost category [17]. An alpha level will be set at 0.05 for all cost analyses. The normality of the HRQoL data will be tested using the Shapiro-Wilk test. In case data is normally distributed a parametric test (independent t-test or ANOVA in case of multiple groups) will be computed. In case data is not normally distributed a nonparametric test (Mann-Whitney *U* or Kruskal-Wallis in case of multiple groups) will be performed. Continuous data will be described as mean (standard deviation) and categorical data will be presented as count (percentage). A p-value ≤.05 will be considered statistically significant. Few missing data is expected due to the restrictions on the online survey. Inconsistencies will be checked and if necessary, an appropriate method for dealing with missing data will be sought. Analyses will be performed using SPSS IBM version 26 and R-software version 4.1.0 (package: eq5d).

### Sensitivity and subgroup analyses

To assess the robustness of the methodological and parametric approach chosen sensitivity analysis will be conducted. To test the methodological uncertainty, sensitivity analyses will be done using the UK-tariff instead of the Dutch tariff and by using the Human Capital Approach (HCA) rather than the Friction Cost Method (FCM) approach to determine productivity losses [19]. To test the influence of alternative perspectives on cost outcomes, costs will be estimated from the healthcare perspective rather than a societal perspective. Furthermore, sub-group analysis will be performed using categories based on the age of the participants to compare the burden of AIS at different time points.

## Discussion

Currently, limited knowledge is available on the burden of disease that adolescent idiopathic scoliosis poses on society. The limited number of studies that report on the burden either exclusively consider costs associated with surgical procedures or assess nonoperative treatment costs in adults rather than adolescents [13], [14], [16]. This study will consider the burden of AIS from a societal perspective, taking not only health care costs into account but also aspects such as family and patient costs, other sectors costs as well as productivity losses. This study will determine the burden of AIS using a survey consisting of 2 costing questionnaires (iMTA-MCQ, iMTA-PCQ) and 2 HRQoL questionnaires (EQ-5D-5L/EQ-5D-Y, SRSr-22). The burden-of-disease estimate that will be derived from this study will be used to raise awareness and wider support for both the informal caretakers and the patient community among healthcare professionals and policymakers. The results obtained in this study may potentially advocate the need for more research on the (cost)- effectiveness treatment or earlier detection screening of AIS.

### Strengths and limitations

The proposed study has several strengths and limitations. A major strength is a standardization and transparency with which the methodological steps are portrayed. The use of reporting guidelines and protocol registration will help ensure research integrity [18], [20]. Additionally, validated questionnaires that look at quality-of-life aspects both generically and disease specifically were used. Furthermore, the costing measurements were performed using the preferred bottom-up approach [37], [38].

However, some limitations are also expected related to the nature of the study design. Due to its cross-sectional design, the impact on quality of life cannot be measured over time. Additionally, a prevalence-based approach was adopted due to practical time constraints, implying that costs can only be estimated for a given period (a year) rather than a lifetime. However, a comparison will be made between different age groups.

Another limitation is that retrospective, self-reported questionnaires can lead to recall bias and inaccuracy of the answers provided. To minimize this a maximum recall period of 3 months was used except for certain questions of the SRSr-22 which refer to the last 6 months. Additionally, due to use of standardized questionnaires no additional information can be obtained via follow-up questions.

Furthermore, to provide an accurate estimate of the burden of AIS for patients and healthcare professionals, broad inclusion criteria were used to include a study population that is representative of the real-world situation. However, the diagnosis of patients cannot be verified using the survey. Also, due to participation on a voluntary basis there is a risk of selection bias. Additionally, measures were taken to prevent multiple submissions by enabling the prevent multiple submissions option in Qualtrics, but this can be circumvented. However, considering the length of the survey and that no incentive will be provided it is unlikely participants will submit multiple responses. Lastly, specific age groups (<15 years old) may be underrepresented due to the required time investment and help required of parents to complete the questionnaire.

### Generalizability

This study protocol may be used as a blueprint for studies in alternative settings. However, considering the differences in healthcare system organization and financing between countries, country-specific analysis is advised.

## Conclusion

This study protocol provides a transparent guideline for conducting a burden of disease study that aims to determine the burden associated with AIS from a societal perspective in the Netherlands. Subsequently, this protocol may serve as a blueprint for future studies in alternative settings. Furthermore, the results of this study are expected to help mitigate discussion regarding prevention such as early detection screening for AIS.

## Ethics approval and consent to participate

Non-WMO approval has been obtained from the Medical Ethical Testing Committee (METC) of the UM/ MUMC+ (METC 2022-3166) as well as consent from the advisory board of the MUMC+. All methods were carried out in accordance with relevant guidelines and regulations.

## Data availability

Data collected throughout this study will be available upon reasonable request.

## Patient informed consent statement

Informed consent was obtained prior to filling in the questionnaire.

## Author's contributions

All authors contributed to the design of the study protocol. TH wrote the manuscript with great contribution from SE, PW, GvM and JA. All authors reviewed the manuscript and approved the final version.

## Declaration of Competing Interest

The authors declare that they have no known competing financial interests or personal relationships that could have appeared to influence the work reported in this paper.

## Appendix Supplementary materials

Image, application 1
