# Supplementary material for: The economic and societal burden associated with adolescent idiopathic scoliosis: A burden-of-disease study protocol
Source: N Am Spine Soc J. 2023 May 18;14:100231. doi: 10.1016/j.xnsj.2023.100231 (PMC10333714; doi:10.1016/j.xnsj.2023.100231)
Supplement: Supplementary file 1 [file mmc1.pdf]

Supplementary materials

I: iMTA Questionnaires

The iMTA-MCQ and iMTA-PCQ questionnaires are available upon request using:

iMTA- Medical Consumption Questionnaire

https://www.imta.nl/questionnaires/imcq/

iMTA- Productivity Cost Questionnaire

https://www.imta.nl/questionnaires/ipcq/

II: EuroQol Questionnaires

Zet bij iedere groep in de lijst hieronder een kruisje in het hokje achter de zin die het beste past bij jouw eigen gezondheid vandaag.

1. **MOBILITEIT**

Ik heb geen problemen met lopen ❑

Ik heb een beetje problemen met lopen ❑

Ik heb matige problemen met lopen ❑

Ik heb ernstige problemen met lopen ❑

Ik ben niet in staat om te lopen ❑

2. **ZELFZORG**

Ik heb geen problemen met mijzelf wassen of aankleden ❑

Ik heb een beetje problemen met mijzelf wassen of aankleden ❑

Ik heb matige problemen met mijzelf wassen of aankleden ❑

Ik heb ernstige problemen met mijzelf wassen of aankleden ❑

Ik ben niet in staat mijzelf te wassen of aan te kleden ❑

3. **DAGELIJKSE ACTIVITEITEN *(bijv. werk, studie, huishouden, gezins- en vrijetijdsactiviteiten)***

Ik heb geen problemen met mijn dagelijkse activiteiten ❑

Ik heb een beetje problemen met mijn dagelijkse activiteiten ❑

Ik heb matige problemen met mijn dagelijkse activiteiten ❑

Ik heb ernstige problemen met mijn dagelijkse activiteiten ❑

Ik ben niet in staat mijn dagelijkse activiteiten uit te voeren ❑

4. **PIJN/ONGEMAK**

Ik heb geen pijn of ongemak ❑

Ik heb een beetje pijn of ongemak ❑

Ik heb matige pijn of ongemak ❑

Ik heb ernstige pijn of ongemak ❑

Ik heb extreme pijn of ongemak ❑

5. **ANGST/SOMBERHEID**

Ik ben niet angstig of somber ❑

Ik ben een beetje angstig of somber ❑

Ik ben matig angstig of somber ❑

Ik ben erg angstig of somber ❑

Ik ben extreem angstig of somber ❑

6. EQ_VAS

De beste gezondheid die je jezelf kunt voorstellen

- We willen weten hoe goed of slecht jouw gezondheid VANDAAG is.
- Deze meetschaal (te vergelijken met een thermometer) loopt van 0 tot 100.
- 100 staat voor de <u>beste</u> gezondheid die je jezelf kunt voorstellen.
  0 staat voor de <u>slechtste</u> gezondheid die je jezelf kunt voorstellen.
- Plaats een kruisje op de meetschaal om aan te geven hoe goed of hoe slecht jouw gezondheid VANDAAG is.
- Noteer het getal waarbij u de X heeft geplaatst in onderstaand vakje.

jouw gezondheid VANDAAG = ☐

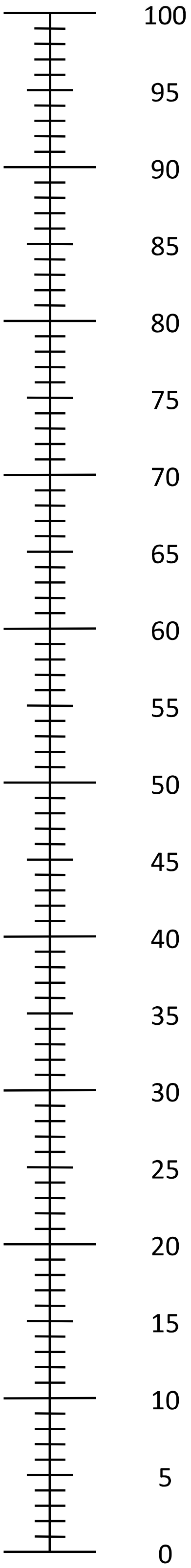

De slechtste gezondheid die je jezelf kunt voorstellen

Health Questionnaire

English version for the UK

Sample

© 2009 EuroQol Research Foundation. EQ-5D™ is a trade mark of the EuroQol Research Foundation. UK (English) v1.2

Under each heading, please tick the ONE box that best describes your health TODAY.

MOBILITY

I have no problems in walking about ❑
I have slight problems in walking about ❑
I have moderate problems in walking about ❑
I have severe problems in walking about ❑
I am unable to walk about ❑

SELF-CARE

I have no problems washing or dressing myself ❑
I have slight problems washing or dressing myself ❑
I have moderate problems washing or dressing myself ❑
I have severe problems washing or dressing myself ❑
I am unable to wash or dress myself ❑

USUAL ACTIVITIES *(e.g. work, study, housework, family or leisure activities)*

I have no problems doing my usual activities ❑
I have slight problems doing my usual activities ❑
I have moderate problems doing my usual activities ❑
I have severe problems doing my usual activities ❑
I am unable to do my usual activities ❑

PAIN / DISCOMFORT

I have no pain or discomfort ❑
I have slight pain or discomfort ❑
I have moderate pain or discomfort ❑
I have severe pain or discomfort ❑
I have extreme pain or discomfort ❑

ANXIETY / DEPRESSION

I am not anxious or depressed ❑
I am slightly anxious or depressed ❑
I am moderately anxious or depressed ❑
I am severely anxious or depressed ❑
I am extremely anxious or depressed ❑

Sample

© 2009 EuroQol Research Foundation. EQ-5D™ is a trade mark of the EuroQol Research Foundation. UK (English) v1.2

- We would like to know how good or bad your health is TODAY.
- This scale is numbered from 0 to 100.
- 100 means the <u>best</u> health you can imagine.
  0 means the <u>worst</u> health you can imagine.
- Please mark an X on the scale to indicate how your health is TODAY.
- Now, write the number you marked on the scale in the box below.

YOUR HEALTH TODAY =

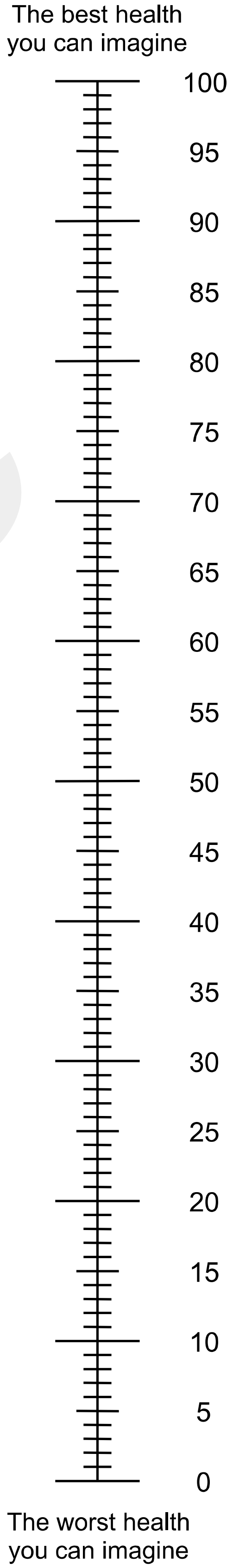

Sample

© 2009 EuroQol Research Foundation. EQ-5D™ is a trade mark of the EuroQol Research Foundation. UK (English) v1.2

Zet bij iedere groep in de lijst hieronder EEN kruisje in het hokje dat het best weergeeft hoe u de gezondheid van het kind VANDAAG zou beschrijven.

1. **Beweging (lopen)**

Hij/zij heeft geen problemen met lopen ❑

Hij/zij heeft een beetje problemen met lopen ❑

Hij/zij heeft veel problemen met lopen ❑

2. **Voor zichzelf zorgen**

Hij/zij heeft geen problemen met wassen of aankleden ❑

Hij/zij heeft een beetje problemen met wassen of aankleden ❑

Hij/zij heeft veel problemen met wassen of aankleden ❑

3. **Dagelijkse activiteiten** ***(bijv. naar school gaan, hobby's, sporten, spelen, naar familie en vrienden gaan)***

Hij/zij heeft geen problemen met zijn/haar dagelijkse activiteiten ❑

Hij/zij heeft een beetje problemen met zijn/haar dagelijkse activiteiten ❑

Hij/zij heeft veel problemen met zijn/haar dagelijkse activiteiten ❑

4. **Pijn of andere klachten**

Hij/zij heeft geen pijn of andere klachten ❑

Hij/zij heeft een beetje pijn of andere klachten ❑

Hij/zij heeft veel pijn of andere klachten ❑

5. **Bezorgd, verdrietig of ongelukkig**

Hij/zij is niet bezorgd, verdrietig of ongelukkig ❑

Hij/zij is een beetje bezorgd, verdrietig of ongelukkig ❑

Hij/zij is erg bezorgd, verdrietig of ongelukkig ❑

6. EQ_VAS

- We willen graag weten hoe goed of slecht u denkt dat de gezondheid van het kind VANDAAG is.
- Deze lijn is genummerd van 0 tot en met 100.
- 100 geeft de beste gezondheid aan die u zich kunt voorstellen.
- 0 geeft de slechtste gezondheid aan die u zich kunt voorstellen.
- Wilt u met een kruisje op de genummerde lijn het punt markeren dat aangeeft hoe goed of slecht u denkt dat de gezondheid van het kind VANDAAG is.
- Noteer het getal waarbij u de X heeft geplaatst in onderstaand vakje.

Gezondheid van het kind VANDAAG =

De beste gezondheid die u zich kunt voorstellen

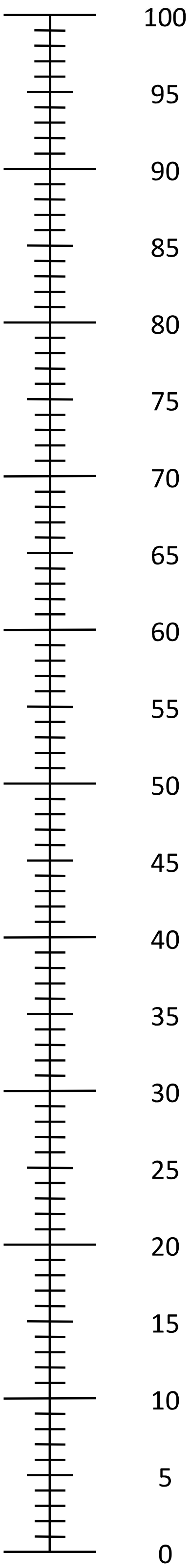

De slechtste gezondheid die u zich kunt voorstellen

Health Questionnaire

English version for the UK

Sample

© 2008 EuroQol Research Foundation. EQ-5D™ is a trade mark of the EuroQol Research Foundation. UK (English) v2.2

Under each heading, please tick the ONE box that best describes your health TODAY.

MOBILITY *(walking about)*

I have no problems walking about ☐
I have some problems walking about ☐
I have a lot of problems walking about ☐

LOOKING AFTER MYSELF

I have no problems washing or dressing myself ☐
I have some problems washing or dressing myself ☐
I have a lot of problems washing or dressing myself ☐

DOING USUAL ACTIVITIES *(for example, going to school, hobbies, sports, playing, doing things with family or friends)*

I have no problems doing my usual activities ☐
I have some problems doing my usual activities ☐
I have a lot of problems doing my usual activities ☐

HAVING PAIN OR DISCOMFORT

I have no pain or discomfort ☐
I have some pain or discomfort ☐
I have a lot of pain or discomfort ☐

FEELING WORRIED, SAD OR UNHAPPY

I am not worried, sad or unhappy ☐
I am a bit worried, sad or unhappy ☐
I am very worried, sad or unhappy ☐

Sample

© 2008 EuroQol Research Foundation. EQ-5D™ is a trade mark of the EuroQol Research Foundation. UK (English) v2.2

- We would like to know how good or bad your health is TODAY.
- This line is numbered from 0 to 100.
- 100 means the best health you can imagine.
  0 means the worst health you can imagine.
- Please mark an X on the line that shows how your health is TODAY.
- Now, write the number you marked on the line in the box below.

YOUR HEALTH TODAY =

The best health
you can imagine

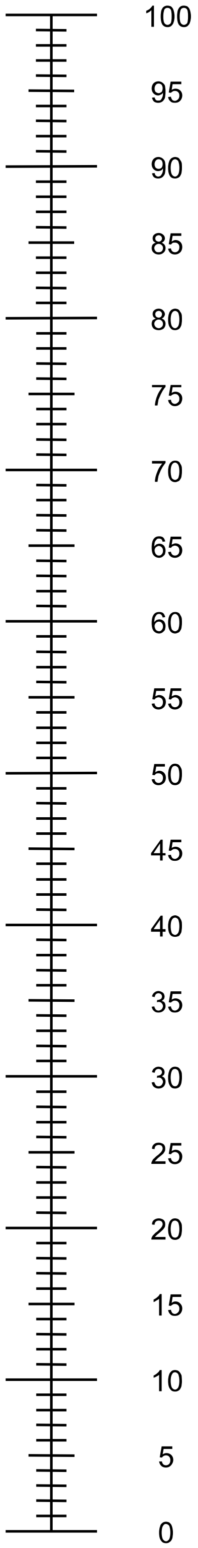

The worst health
you can imagine

Sample

© 2008 EuroQol Research Foundation. EQ-5D™ is a trade mark of the EuroQol Research Foundation. UK (English) v2.2

Vragenlijst voor scoliosepatiënten (SRS-22r)

Achternaam: ________________________________

Eerste letter voornaam: _____

Geslacht: ☐ Man ☐ Vrouw

Geboortedatum: Dag _ _ maand _ _ jaar _ _ _ _

Het is belangrijk dat je zelf de vragenlijst invult.
Er zijn geen goede of foute antwoorden.
Beantwoord alsjeblieft alle vragen.
Voor alle vragen geldt dat je maar één antwoord mag aankruisen; zie het voorbeeld hiernaast.

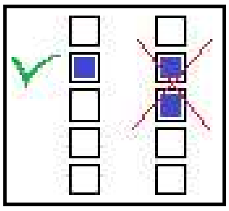

1. **Welke van de volgende mogelijkheden beschrijft het beste de hoeveelheid pijn die je hebt gehad <u>in de afgelopen 6 maanden</u>?**
   ☐ Geen pijn
   ☐ Een beetje pijn
   ☐ Matige pijn
   ☐ Matig tot veel pijn
   ☐ Veel pijn

2. **Welke van de volgende mogelijkheden beschrijft het beste de hoeveelheid pijn die je hebt gehad <u>in de afgelopen maand</u>?**
   ☐ Geen pijn
   ☐ Een beetje pijn
   ☐ Matige pijn
   ☐ Matig tot veel pijn
   ☐ Veel pijn

3. **Ben je in de afgelopen 6 maanden erg gespannen geweest?**
   ☐ Nooit
   ☐ Bijna nooit
   ☐ Af en toe
   ☐ Meestal
   ☐ Altijd

4. **Hoe zou je je voelen als de vorm van je rug de rest van je leven blijft, zoals die nu is?**
   ☐ Zeer tevreden
   ☐ Een beetje tevreden
   ☐ Niet tevreden, maar ook niet ontevreden
   ☐ Een beetje ontevreden
   ☐ Zeer ontevreden

5. **Wat is jouw huidige niveau van lichamelijke activiteiten?**
   - ☐ Bedlegerig/rolstoel
   - ☐ Voornamelijk geen activiteiten
   - ☐ Lichte werkzaamheden en lichte sport, zoals huishoudelijke klusjes en wandelen
   - ☐ Gemiddelde werkzaamheden en gemiddeld intensieve sporten, zoals joggen en fietsen
   - ☐ Alle activiteiten zonder beperkingen

6. **Hoe vind je jezelf eruitzien met kleren aan?**
   - ☐ Zeer goed
   - ☐ Goed
   - ☐ Redelijk
   - ☐ Slecht
   - ☐ Zeer slecht

7. **Heb je in de afgelopen 6 maanden het gevoel gehad dat je zo diep in de put zat, dat niets je kon opvrolijken?**
   - ☐ Zeer vaak
   - ☐ Vaak
   - ☐ Af en toe
   - ☐ Bijna nooit
   - ☐ Nooit

8. **Heb je rugpijn wanneer je rust?**
   - ☐ Zeer vaak
   - ☐ Vaak
   - ☐ Soms
   - ☐ Bijna nooit
   - ☐ Nooit

9. **Wat is je huidige niveau van werk- of schoolactiviteiten?**
   - ☐ Helemaal niet beperkt
   - ☐ Een beetje beperkt
   - ☐ Ernstig beperkt
   - ☐ Zeer ernstig beperkt
   - ☐ Totaal beperkt

10. **Welke van de volgende mogelijkheden beschrijft het beste hoe je romp eruitziet? Onder romp verstaan we het menselijk lichaam met uitzondering van het hoofd, armen en benen.**
    - ☐ Zeer goed
    - ☐ Goed
    - ☐ Redelijk
    - ☐ Slecht
    - ☐ Zeer slecht

11. Welke van de volgende mogelijkheden beschrijft het beste je gebruik van pijnmedicatie voor je rug?

- ☐ Geen pijnstillers
- ☐ Wekelijks of minder vaak, lichte pijnstillers (bijvoorbeeld paracetamol, ibuprofen of aspirine)
- ☐ Dagelijks lichte pijnstillers (bijvoorbeeld paracetamol, ibuprofen of aspirine)
- ☐ Wekelijks of minder vaak, zware pijnstillers (bijvoorbeeld diclofenac of morfine)
- ☐ Dagelijks zware pijnstillers (bijvoorbeeld diclofenac of morfine)

12. Beperkt je rug je mogelijkheden om dingen te doen in en rond het huis?

- ☐ Nooit
- ☐ Bijna nooit
- ☐ Soms
- ☐ Vaak
- ☐ Zeer vaak

13. Heb je je kalm en rustig gevoeld gedurende de afgelopen 6 maanden?

- ☐ Altijd
- ☐ Meestal
- ☐ Soms
- ☐ Bijna nooit
- ☐ Nooit

14. Heb je het gevoel dat je rugaandoening je persoonlijke relaties beïnvloedt?

- ☐ Niet
- ☐ Nauwelijks
- ☐ Een beetje
- ☐ Veel
- ☐ Heel veel

15. Heb jij of heeft je familie financiële problemen door jouw rugaandoening?

- ☐ Heel veel
- ☐ Veel
- ☐ Een beetje
- ☐ Nauwelijks
- ☐ Geen

16. Heb je je in de afgelopen 6 maanden somber gevoeld?

- ☐ Nooit
- ☐ Bijna nooit
- ☐ Soms
- ☐ Vaak
- ☐ Zeer vaak

17. Hoeveel dagen heb je in de afgelopen 3 maanden van werk, huishoudelijk werk of school verzuimd vanwege rugpijn?

- ☐ 0 dagen
- ☐ 1 dag
- ☐ 2 dagen
- ☐ 3 dagen
- ☐ 4 dagen of meer

18. Beperkt je rugaandoening je in dingen ondernemen met familie of vrienden?

- ☐ Nooit
- ☐ Bijna nooit
- ☐ Soms
- ☐ Vaak
- ☐ Zeer vaak

19. Voel je jezelf aantrekkelijk met de huidige toestand van je rug?

- ☐ Ja, heel erg aantrekkelijk
- ☐ Ja, een beetje aantrekkelijk
- ☐ Niet aantrekkelijk, maar ook niet onaantrekkelijk
- ☐ Nee, niet erg aantrekkelijk
- ☐ Nee, helemaal niet aantrekkelijk

20. Ben je een opgewekt persoon geweest in de afgelopen 6 maanden?

- ☐ Nooit
- ☐ Bijna nooit
- ☐ Soms
- ☐ Meestal
- ☐ Altijd

21. Ben je tevreden met het resultaat van de controles en behandeling van je rug tot nu toe?

- ☐ Erg tevreden
- ☐ Tevreden
- ☐ Niet tevreden, maar ook niet ontevreden
- ☐ Ontevreden
- ☐ Erg ontevreden

22. Zou je weer voor hetzelfde beleid kiezen als je dezelfde aandoening had?

- ☐ Zeker wel
- ☐ Waarschijnlijk wel
- ☐ Ik weet het niet zeker
- ☐ Waarschijnlijk niet
- ☐ Zeker niet

- ✔ *Alles ingevuld? Eén antwoord aangekruist per vraag?*
- ✔ ***Dank voor het invullen!***

<u>**SRS-22r** Patient Questionnaire</u>

Patient Name: ____________ ________ ______________ Date of Birth:
First MI Last Mo Day Yr

Today's Date: ______ ________ Age: --- +_______

Mo Day Yr Yrs Mo

Medical Record #: ___________________

INSTRUCTIONS: We are carefully evaluating the condition of your back and it is **IMPORTANT THAT YOU ANSWER EACH OF THESE QUESTIONS YOURSELF.** Please **CIRCLE THE <u>ONE BEST</u> <u>ANSWER</u> TO EACH QUESTION.**

1. Which one of the following best describes the amount of pain you have experienced during the past 6 months?

   None
   Mild
   Moderate
   Moderate to severe
   Severe

2. Which one of the following best describes the amount of pain you have experienced over the last month?

   None
   Mild
   Moderate
   Moderate to severe
   Severe

3. During the past 6 months have you been a very nervous person?

   None of the time
   A little of the time
   Some of the time
   Most of the time
   All of the time

(CONTINUED ON NEXT PAGE)

4. If you had to spend the rest of your life with your back shape as it is right now, how would you feel about it?

Very happy
Somewhat happy
Neither happy nor unhappy
Somewhat unhappy
Very unhappy

5. What is your current level of activity?

Bedridden
Primarily no activity
Light labor and light sports
Moderate labor and moderate sports
Full activities without restriction

6. How do you look in clothes?

Very good
Good
Fair
Bad
Very bad

7. In the past 6 months have you felt so down in the dumps that nothing could cheer you up?

Very often
Often
Sometimes
Rarely
Never

8. Do you experience back pain when at rest?

Very often
Often
Sometimes
Rarely
Never

9. What is your current level of work/school activity?

100% normal
75% normal
50% normal
25% normal
0% normal

(CONTINUED ON NEXT PAGE)

10. Which of the following best describes the appearance of your trunk; defined as the human body except for the head and extremities?

Very good
Good
Fair
Poor
Very Poor

11. Which one of the following best describes your pain medication use for back pain?

None
Non-narcotics weekly or less (e.g., aspirin, Tylenol, Ibuprofen)
Non-narcotics daily
Narcotics weekly or less (e.g. Tylenol III, Lorcet, Percocet)
Narcotics daily

12. Does your back limit your ability to do things around the house?

Never
Rarely
Sometimes
Often
Very Often

13. Have you felt calm and peaceful during the past 6 months?

All of the time
Most of the time
Some of the time
A little of the time
None of the time

14. Do you feel that your back condition affects your personal relationships?

None
Slightly
Mildly
Moderately
Severely

(CONTINUED ON NEXT PAGE)

15. Are you and/or your family experiencing financial difficulties because of your back?

Severely
Moderately
Mildly
Slightly
None

16. In the past 6 months have you felt down hearted and blue?

Never
Rarely
Sometimes
Often
Very often

17. In the last 3 months have you taken any days off of work, including household work, or school because of back pain?

0 days
1 day
2 days
3 days
4 or more days

18. Does your back condition limit your going out with friends/family?

Never
Rarely
Sometimes
Often
Very often

19. Do you feel attractive with your current back condition?

Yes, very
Yes, somewhat
Neither attractive nor unattractive
No, not very much
No, not at all

20. Have you been a happy person during the past 6 months?

None of the time
A little of the time
Some of the time
Most of the time
All of the time

(CONTINUED ON NEXT PAGE)

21. Are you satisfied with the results of your back management?

- Very satisfied
- Satisfied
- Neither satisfied nor unsatisfied
- Unsatisfied
- Very unsatisfied

22. Would you have the same management again if you had the same condition?

- Definitely yes
- Probably yes
- Not sure
- Probably not
- Definitely not

Thank you for completing this questionnaire. Please comment if you wish.

END
